# Impact of an Integrated Yoga Therapy Protocol on Insulin Resistance and Glycemic Control in Patients with Type 2 Diabetes Mellitus

**DOI:** 10.5041/RMMJ.10462

**Published:** 2022-01-27

**Authors:** Manoharan Mangala Gowri, Jayanthi Rajendran, Abu Raghavan Srinivasan, Ananda Balayogi Bhavanani, Ramanathan Meena

**Affiliations:** 1 Department of Biochemistry, Mahatma Gandhi Medical College & Research Institute, Sri Balaji Vidyapeeth (SBV), SBV Campus, Pillaiyarkuppam, Pondicherry, India; 2 Centre for Yoga Therapy Education and Research, Sri Balaji Vidyapeeth (SBV), SBV Campus, Pillaiyarkuppam, Pondicherry, India

**Keywords:** DM, dyslipidemia, HOMA-IR, yoga therapy

## Abstract

**Objective:**

Diabetes mellitus (DM), characterized by chronic hyperglycemia, is attributed to relative insulin deficiency or resistance, or both. Studies have shown that yoga can modulate parameters of insulin resistance. The present study explored the possible beneficial effects of integrated yoga therapy with reference to glycemic control and insulin resistance (IR) in individuals with diabetes maintained on standard oral medical care with yoga therapy, compared to those on standard oral medical care alone.

**Methods:**

In this study, the subjects on yoga intervention comprised 35 type 2 diabetics, and an equal number of volunteers constituted the control group. Subjects ranged in age from 30 to 70 years, with hemoglobin A1c (HbA1c) test more than 7%, and were maintained on diabetic diet and oral hypoglycemic agents. Blood samples were drawn prior to and after 120 days of integrated yoga therapy intervention. Fasting blood glucose (FBG), post-prandial blood glucose (PPBG), HbA1c, insulin, and lipid profile were assessed in both the intervention and control groups.

**Results:**

The intervention group revealed significant improvements in body mass index (BMI) (0.7 kg/m^2^ median decrease; *P*=0.001), FBG (20 mg/dL median decrease; *P*<0.001), PPBG (33 mg/dL median decrease; *P*<0.001), HbA1c (0.4% median decrease; *P*<0.001), homeostatic model assessment for insulin resistance (HOMA-IR) (1.2 median decrease; *P*<0.001), cholesterol (13 mg/dL median decrease, *P*=0.006), triacylglycerol (22 mg/dL median decrease; *P*=0.027), low-density lipoprotein (6 mg/dL median decrease; *P*=0.004), and very-low-density lipoprotein levels (4 mg/dL median decrease; *P*=0.032). Increases in high-density lipoprotein after 120 days were not significant (6 mg/dL median increase; *P*=0.15). However, when compared to changes observed in patients in the control group, all these improvements proved to be significant.

**Conclusion:**

Administration of integrated yoga therapy to individuals with diabetes leads to a significant improvement in glycemic control, insulin resistance, and key biochemical parameters.

## INTRODUCTION

Diabetes mellitus (DM) is a leading cause of mortality worldwide. As per 2019 estimates, 77 million individuals had diabetes in India. The figures are expected to touch a staggering 134 million by the year 2045.^1^

Yoga has its roots firmly entrenched in India and evolved over the last 4,000 years as a traditional form of mind–body training. Yoga *asanas* (typical postures) and *pranayama* (breath control) have recently become very popular, and the role of yoga in several chronic diseases has become the topic of current interest.^2^–^4^

A host of evidence-based trials has shown that yoga can pronouncedly attenuate fasting blood glucose and improve glycemic control, besides improving the lipid levels and quality of life in diabetic paients.^5^–^12^ Mind–body practices essentially depend on the ability of the mind to enhance physical health (and vice versa). In recent years, the practice of yoga as a viable non-invasive approach has been steadily rising, thanks to evidence-based reports.^13^–^15^ It must be stressed that yoga is generally safe and simple to learn and could be practiced by individuals who are interested in yoga learning.^16^–^19^ Furthermore, yoga paves the way for nodal strategies to combat stress, reduce anxiety, and enhance positive emotions.^20^ Several earlier findings indicate that yogic practices may lead to pronounced improvements in DM management. The significance of non-drug lifestyle modification could hold the all-important and decisive key to diabetic management. In this context there is a need to educate patients and their families on the benefits of evidence-based yoga therapy.^21^,^22^ The main objective of this study was to evaluate the added value of an integrated yoga therapy protocol in improving fasting blood glucose (FBG), post-prandial blood glucose (PPBG), hemoglobin A1c (HbA1c), insulin, and lipid profile in type 2 diabetic patients treated with oral hypoglycemics.

## METHODS

### Setting

This study was carried out over two years (2018–2020) by the Department of Biochemistry, the Diabetic Outpatient Clinic, and the Yoga Therapy Centre of Mahatma Gandhi Medical College and Research Institute, Sri Balaji Vidyapeeth (SBV), Puducherry, India, a tertiary care teaching hospital. The study was approved by the institutional research advisory committee and the institutional ethics committee (*vide* project no. Ph.D. Project/2017/05/04 dt. 4/5/2017). All subjects recruited to this study signed an informed consent.

### Study Population

The eligibility assessment was carried out on 159 subjects of both sexes who had visited our diabetic clinics. Among them, 50 patients were excluded since they did not meet the inclusion criteria or had declined to participate for personal reasons. Based on randomization 59 patients were assigned to yoga intervention and 50 served as the controls. Thirty-eight members of the intervention group completed 10 sessions of integrated yoga therapy; 21 were unable to meet the compliance requirements. Of those who had completed ten sessions (*n*=38), 2 were lost at follow-up and 1 relocated due to new employment; these 3 participants were deemed as discontinued. In the control group (*n*=50), 8 were lost during follow-up. Of the remaining 42, 35 were taken to facilitate an exact match in numbers with reference to the yoga group subjects. The sexes of the members of both groups were also noted (yoga intervention: females, *n*=21, males, *n*=14; control: females, *n*=12, males, *n=23*).

The final study group included males and females between 30 and 70 years of age. Care was taken to include only those with a type 2 DM (T2D) disease duration of a minimum of three years and HbA1c more than 7%. All had to be on a diabetic diet, and maintained on medical oral hypoglycemic agents. Potential recruits had to agree to participate in a randomized study in which only some of the study subjects would be asked to participate in yoga therapy sessions.

Potential recruits were excluded if they had type 1 DM or conditions associated with insulin resistance, such as pregnancy or nursing, or were smokers, tobacco chewers, and alcohol consumers. Subjects who had expressed inability to perform the yoga techniques as given in the protocol were also promptly excluded from the study.

### Randomization

Using a computer-generated random allocation sequence, study participants were assigned to one of two groups. Both groups were treated by conventional treatment for T2D: diabetic diet and medical oral hypoglycemic drugs. Patients in Group I (study group) were treated by the conventional treatment for T2D as described above, and were also asked to participate in an integrated yoga therapy intervention. Patients in Group II served as controls.

### Study Intervention

Intervention consisted of 10 sessions essentially based on twice weekly yoga therapy taught initially in a graded manner. Patients in Group I were also asked to practice at home twice weekly, to maintain the regularity, rhythm, and repetition that are essential to the practice. Otherwise, the study subjects in both Group I and Group II were advised to maintain their regimen of oral hypoglycemic agents. Regular phone calls were made by the principal investigator of the study to subjects in order to facilitate compliance *in toto*. The study subjects as well as the controls were advised to return to the outpatient department following a period of 120 days.

Baseline investigations were performed and blood samples obtained after 120 days. Proper advice regarding diet and lifestyle modification was duly provided. Both groups were advised to take fiber-rich foods such as leafy green vegetables. The yoga-based intervention protocol is detailed in Table 1, with suggested benefits for each posture.

**Table 1:** Yoga Therapy Protocol for Individuals with Type 2 Diabetes Mellitus (DM).^23^

Yoga Therapy	Duration	Benefits
***Jathis*** ** (warming up)**	5 min	Helps loosen the joints and prepares the body for performing *asanas*

**Standing postures**	Total 10 min	
Palm tree posture: *Talasana* Triangle posture: *Trikonasana* Extended side angle posture: *Parsvakonasana* Hero pose: *Veerasana*	20 sec each side/*asanas*/3 sets	Stretches and tones up all back and leg muscles Relieves upper and lower extremities stiffness Helps reduce the fat accumulation around the waist and hip regions, which helps in insulin sensitivity by ameliorating insulin resistance

**Sitting postures**	Total 7 min for all sitting *asanas*	
Wind-relieving pose: *Pavanamukthasana*	10 sec/5 rounds	Enhances digestion and elimination of waste Helps reduce fat accumulation around the abdomen, waist, and hip regions, thus reshaping the body structure
Compact pose drawing everything to one’s center: *Navasana*	10 sec/5 rounds	Tones up abdominal and thigh muscles in a healthy manner Sends a pronounced blood supply to the pelvic area by creating healthy tension Effective for disorders of the intestines, pancreas, liver, gall bladder, and spleen Stretches and tones up all of the back, arm, and leg muscles This posture gives an excellent massage to the abdominal organs and is useful for DM patients
Half spinal twist pose: *Vakrasana*	10 sec/each side/5 rounds	Helps reduce fat accumulation around the abdomen, waist, and hip region, thus reshaping body structure and helping to fight obesity; these *asanas* also improve insulin sensitivity
Half Lord of the fishes pose: *Ardha matseyendrasana*	10 sec/each side /5 rounds	Helps correct structural deformities of the spine, shoulder, and upper back regions

***Mudras* (gestures)** Topsy-turvy gesture: Viparitakarani*	30 sec/ 30 sec relaxation	Promotes healthy metabolic function by stimulating the pancreas and insulin uptake by the insulin-sensitive body cells (i.e. adipose tissue, heart muscle, and skeletal muscle) Highly recommended for control of blood sugar in DM and corrects thyroid imbalance

***Pranayama* (breathing techniques in the sitting posture)** Moon breathing (left side nostril breathing): *Chandra nadi* Primordial chant: *Pranava* Skull shining breathing technique: *Kapalbhati*	Total 20 min	Tones up cardio-respiratory function that may be compromised in DM Practicing 3–9 rounds daily helps relax the body-emotion-mind complex; this complex enhances insulin sensitivity and decreases insulin sensitivity

**Meditation**	Total 12 min	
*Primordial chant with gesture: Om japa* with *mudras*	5 min	Relaxes the body-emotion-mind complex and provides complete healing through the production of healing vibrations at all levels of existence
Dynamic body relaxation: *Kaya kriya*	6 rounds	Mind and body relaxation
Part by part body relaxation technique: *Marmanasthana kriya*	8 min	

**Relaxation**	5 min	Mind and body relaxation
Corpse pose: *Shavasana*		

**Total**	60 min	

Jathi(s) are warming-up exercises; Asana(s) are characteristic body postures; Mudra(s) are symbolic gestures; Om japa signifies the chanting of sacred sound; Pranayama are breathing techniques.

* To be done with caution.

DM, diabetes mellitus; IR, insulin resistance; min, minute(s); sec, second(s).

### Evaluation of Endpoints

**Figure 1:**
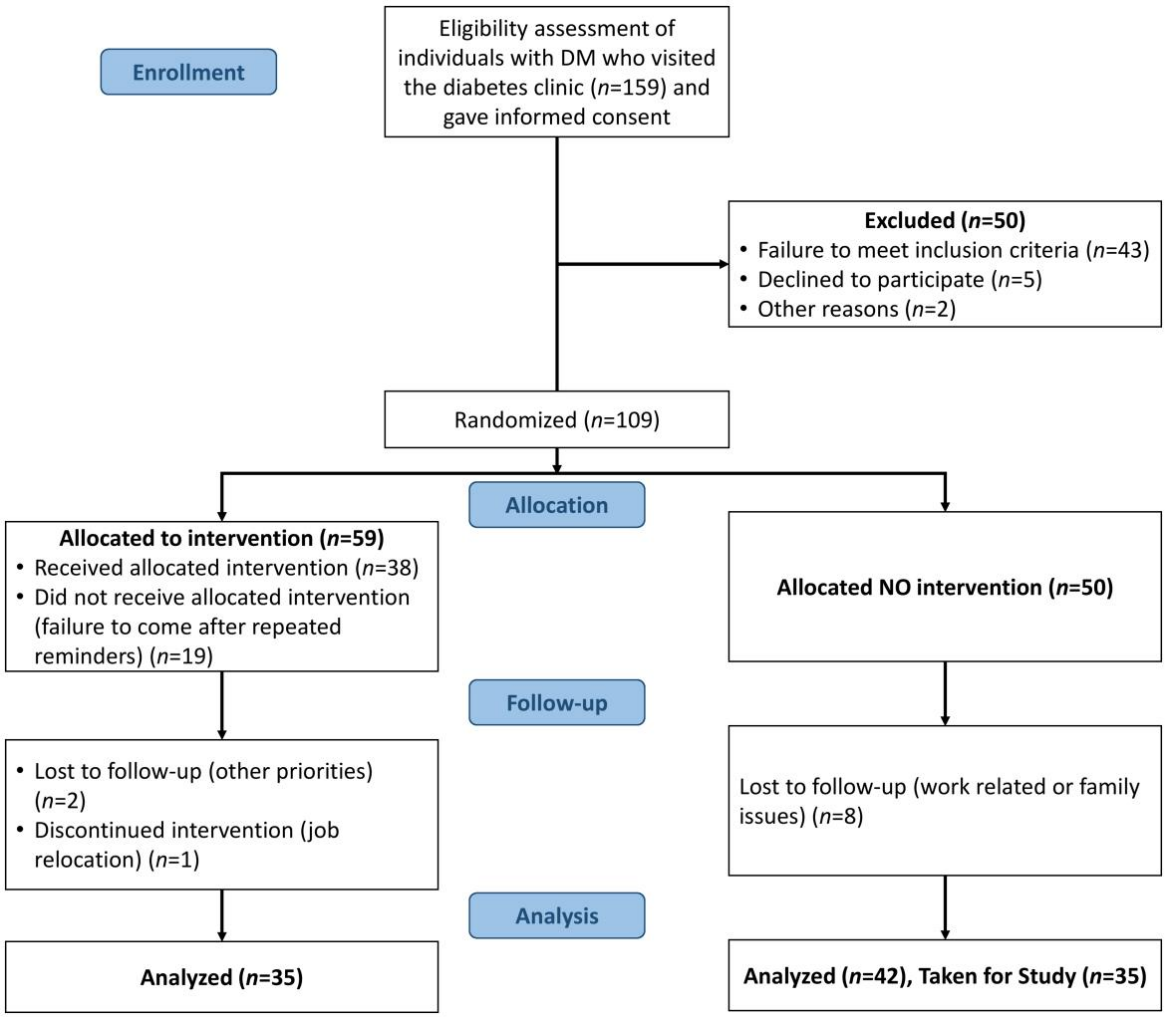
Study Methodology (CONSORT Flow Chart Diagram). DM, diabetes mellitus.

Anthropometric measurements were taken in association with baseline biochemical parameters. The anthropometric measurements and biochemical parameters were also measured after 120 days. Detailed methodology of the study is given in the CONSORT diagram (Figure 1). The fasting or post-absorptive blood sample (12-h) was obtained through a venipuncture in the arm with the individual maintained in an upright position and following 5 minutes in the resting state. All participants were asked to provide one more blood sample to facilitate the quantitation of post-prandial blood glucose, 2 hours after a meal.

Various biochemical parameters were evaluated at baseline and after 120 days for all participants of both groups, including fasting and PPBG, plasma insulin, and lipid profile. Biochemical measurements were based on established laboratory procedures in compliance with the guidelines of the International Federation of Clinical Chemistry and Laboratory Medicine (IFCC).

Fasting and PPBG levels were estimated according to the enzymatic (glucose oxidase-peroxidase) method. Fasting insulin (venous plasma) was estimated by automated electrochemiluminescence. Glycosylated hemoglobin (HbA1c) was determined by high-performance liquid chromatography. Insulin resistance was computed by the homeostatic model assessment computed using the established formula.^24^ Triacylglycerols and total serum cholesterol were quantitated by the enzymatic method. High-density lipoprotein (HDL) cholesterol was quantitated by polyanion precipitation. The LDL cholesterol was computed by the Friedewald equation. Care was taken with reference to the limitations of the Friedewald equation. All subjects underwent follow-up in order to ensure compliance.

The evaluation of biochemical parameters was based on stringent quality control (QC). Internal quality assessment was enabled with the assistance of QC samples provided by M/S Bio-Rad (Hercules, CA, USA). External quality assessment was initiated through a collaborative effort with the Clinical Biochemistry Laboratory (accredited by NABL under ISO/IEC 15189), Christian Medical College, Vellore, Tamilnadu, India which is under the aegis of the Association of Clinical Biochemists of India (ACBI).

### Statistical Analysis

The JASP 0.8.5 software was employed in this study. Differences in baseline characteristics and baseline investigations were compared between Group I and Group II. Differences observed between baseline and 120 days were evaluated for each variable within each group. Finally, Group I and Group II were compared for changes observed between baseline and 120 days. Non-parametric tests were used throughout. A *P* value of<0.05 was considered significant.

## RESULTS

Table 2 presents the baseline characteristics and baseline investigations of both groups. Apart from differences in BMI and PPBG, no other differences were noted between the two groups. Table 3 presents the median and interquartile ranges for each variable in Group I and Group II at baseline and after 120 days. Patients treated with yoga demonstrated significant improvements in BMI, FBG, PPBG, HbA1c, HOMA-IR, cholesterol, TAG, LDL, and VLDL levels; increases in HDL after 120 days were not significant. However, when compared to patients in Group II, all these improvements proved to be significant.

**Table 2:** Baseline Characteristics of the Yoga Group (Group I) and the Control Group (Group II).

Characteristic	Group I Median (IQR)	Group II Median (IQR)	*P* Value
Age (years)	54 (45, 58)	52.5 (46.8, 58)	0.76
BMI (kg/m^2^)	26.6 (25.0, 29.2)	24.6 (22.4, 27.7)	0.028
FBG (mg/dL)	146 (120, 220)	171 (138, 207)	0.33
PPBG (mg/dL)	250 (193, 295)	293 (232, 349)	0.034
HbA1c (%)	8.1 (7.5, 10.3)	8.1 (7.8, 10.1)	0.50
Insulin (μIU/mL)	13.8 (7.2, 19.7)	13.2 (6.6, 22.7)	0.88
HOMA-IR*	5.2 (2.5, 7.5)	5.8 (3.8, 7.2)	0.59
Cholesterol (mg/dL)	193 (148, 217)	190 (149, 211)	0.72
TAG (mg/dL)	114 (80, 157)	114 (97, 173)	0.82
HDL (mg/dL)	51 (45, 58)	55 (47, 60)	0.36
LDL (mg/dL)	116 (80, 145)	108 (77, 131)	0.36
VLDL (mg/dL)	23 (16, 31)	23 (19, 35)	0.84

* HOMA-IR is a computed value and has no units.

BMI, body mass index; FBG, fasting blood glucose; HbA1c, glycosylated hemoglobin; HDL, high-density lipoprotein; HOMA-IR, homeostatic model assessment of insulin resistance ([glucose (mmol/L) × insulin (μIU/mL)] / 22.5); IQR, interquartile range; LDL, low-density lipoprotein; PPBG, post-prandial blood glucose; TAG, triacylglycerol; VLDL, very-low-density lipoprotein.

**Table 3:** Changes Observed Over Time Within the Yoga Group (Group I) and the Control Group (Group II).

Variables	Group I Median (IQR)				Group II Median (IQR)				*P* Value for Changes between Group I and II
	Baseline	Day 120	Change within Group I	*P* value, Changes within Group I	Baseline	Day 120	Change within Group II	*P* Value, Changes within Group II	
BMI (kg/m^2^)	26.6 (25.0, 29.2)	26.0 (24.4, 28.6)	−0.7 (−0.9, 0)	0.001	24.6 (22.4, 27.7)	25.3 (23.4, 28.1)	+0.9 (0.4, 1.8)	0.003	<0.001
FBG (mg/dL)	146 (120, 220)	131 (111, 178)	−20 (−58, −8)	<0.001	171 (138, 207)	198 (158, 270)	+15 (3, 37)	<0.001	<0.001
PPBG (mg/dL)	250 (193, 295)	190 (154, 242)	−33 (−55, −9)	<0.001	293 (232, 349)	299 (263, 389)	+55 (27, 78)	0.008	<0.001
HbA1c (%)	8.1 (7.5, 10.3)	7.5 (6.8, 8.9)	−0.4 (−0.6, −0.2)	<0.001	8.1 (7.8, 10.1)	8.9 (7.9, 10.6)	+0.7 (0.4, 1.7)	<0.001	<0.001
Insulin (μIU/mL)	13.8 (7.2, 19.7)	11.2 (7.5, 16.1)	−1.7 (−3.3, −0.7)	0.017	13.2 (6.6, 22.7)	14.4 (7.3, 23.5)	+1.6 (−0.1, 4.8)	0.019	<0.001
HOMA-IR*	5.2 (2.5, 7.5)	4.0 (2.4, 5.8)	−1.2 (−2.9, −0.4)	<0.001	5.8 (3.8, 7.2)	7.1 (4.3, 10.6)	+1.3 (0.3, 2.7)	0.001	<0.001
Cholesterol (mg/dL)	193 (148, 217)	180 (132, 202)	−13 (−39, 11)	0.006	190 (149, 211)	200 (166, 223)	+10 (0, 35)	0.138	0.002
TAG (mg/dL)	114 (80, 157)	97 (80, 130)	−22 (−50, 0)	0.027	114 (97, 173)	137 (103, 205)	+15 (−10, 40)	0.005	<0.001
HDL (mg/dL)	51 (45, 58)	54 (48, 62)	+6 (−1, 10)	0.15	55 (47, 60)	49 (46, 52)	−5 (−10, 3)	0.019	0.006
LDL (mg/dL)	116 (80, 145)	99 (71, 119)	−6 (−34, 22)	0.004	108 (77, 131)	118 (76, 148)	+15 (0, 34)	0.36	0.012
VLDL (mg/dL)	23 (16, 31)	19 (16, 26)	−4 (−10, 0)	0.032	23 (19, 35)	27 (20, 141)	+3 (−2, 8)	0.002	<0.001

* HOMA-IR is a computed value and has no units.

BMI, body mass index (kg/m^2^); FBG, fasting blood glucose (mg/dL); HbA1c, glycosylated hemoglobin (%); HDL, high-density lipoprotein (mg/dL); HOMA-IR, homeostatic model assessment of insulin resistance ([glucose (mmol/L) × insulin (μIU/mL)] / 22.5); IQR, interquartile range; LDL, low-density lipoprotein (mg/dL); PPBG, post-prandial blood glucose (mg/dL); TAG, triacylglycerol (mg/dL); VLDL, very-low density lipoprotein (mg/dL).

## DISCUSSION

Our study was based on a structured integrated yoga protocol (*Talaasana*, *Trikonasana*, *Parsvakonasana*, *Veerasana*, and *Pavanamukthasana*), and showed that participants had significant reduction in serum total cholesterol, TAG, and LDL cholesterol, as supported by another Indian study. *Ardha matseyendrasana*, *vakrasana*, and *navasana* stimulate the pancreas for insulin secretion.^25^

Yoga has been increasingly considered to be a promising, cost-effective, and non-invasive option in the management of DM, with data from several studies implying that yoga and other mind–body therapeutic modalities could pronouncedly attenuate stress-related hyperglycemia and have a positive effect on blood glucose control. Stress management has become increasingly important in recent times and holds the key to the management of DM. It is believed that *pranayama* techniques and yoga therapy alleviate stress-induced hyperglycemia. The practice of *chandra nadi pranayama*, *pranava pranayama*, and *dhayana* as well as *Om japa* with *mudras* reduces stress and helps in proper metabolism. During periods of stress or trauma, the increased release of epinephrine in conjunction with glucagon leads to increased blood glucose.^26^,^27^ *Kaya kriya* and *marmansthana kriya* are relaxation techniques help to relax the mind and body.

### Mechanism

**Figure 2:**
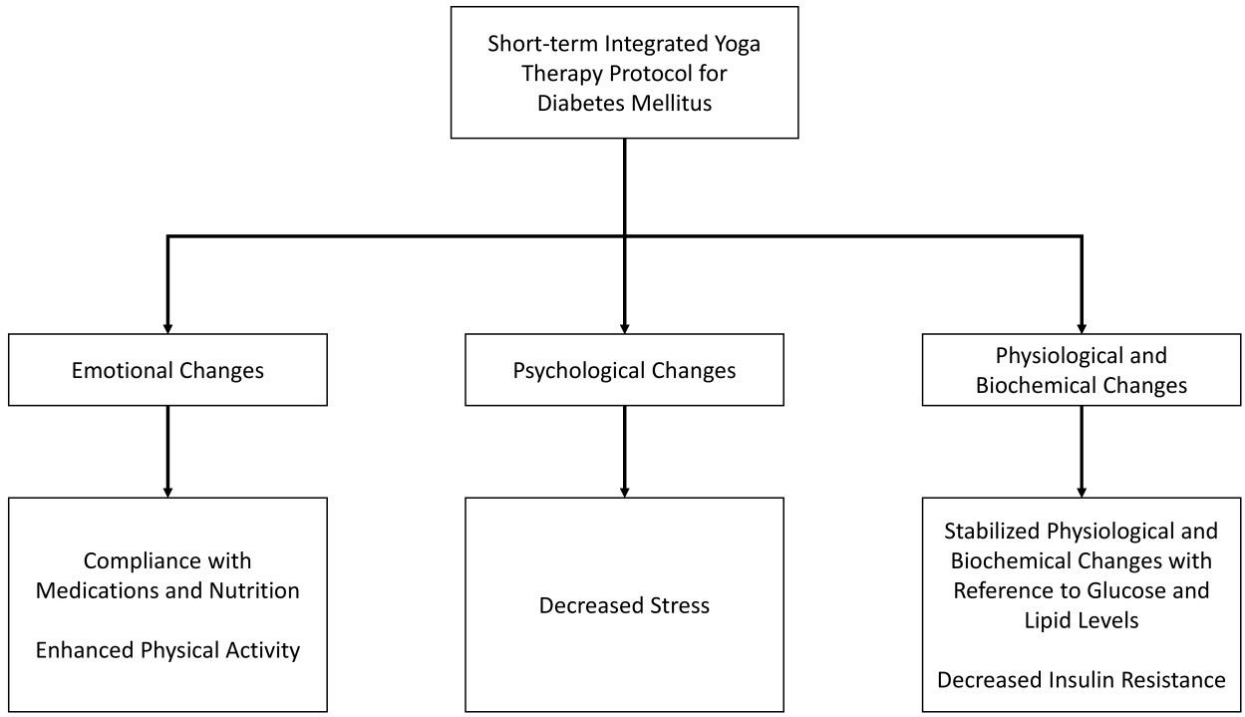
Positive Benefits of Integrated Yoga Therapy Achieved in Patient with Type 2 Diabetes (Group I).

Using controlled breathing techniques, meditation, and different body postures, yoga and other associated programs train the participants to invoke a relaxation response. This response enables the regulation of cortisol and other stress hormones that normally increase blood pressure and blood glucose levels. Enough evidence is available to state that insulin resistance plays a significant pathophysiologic role in DM, in addition to being a risk factor for the development of cardiovascular disease.^28^ The present study examined the effects of practicing a given protocol of 10 twice-weekly guided integrated yoga therapy sessions in individuals with DM over a period of four months’ yoga. The emotional, psychological, physiological, and biochemical changes noted in these patients are outlined in Figure 2. The practice of yoga in these patients resulted in a significant decrease in BMI, body weight, total cholesterol, triacylglycerol, and LDL cholesterol, and an increase in HDL. Earlier, studies had depicted that lifestyle modification and oral drugs could improve diabetic dyslipidemia.^27^ Moreover, insulin resistance (IR) is correlated with a decreased response to the metabolic actions of insulin, including insulin-stimulated glucose disposal and inhibition of hepatic glucose output. The hepatic output of glucose, through gluconeogenesis, has been the subject of much research in recent years and is the favored target for oral hypoglycemic drugs in recent years.^29^ Our results suggest that, compared with controls, intervention through yoga could help improve insulin sensitivity in DM. It must, however, be noted that dynamic measures of insulin sensitivity resemble stimulated insulin action and denote the peripheral insulin-mediated glucose uptake. Existing studies have found positive effects from physical exercise and integrated yoga protocols.^30^ A structured yoga therapy protocol is an additional interventional strategy to improve glycemic control, which is presently recommended to reduce IR seen in DM. The BMI is correlated with IR, visceral fat, fasting blood sugar, and musculoskeletal mass among T2D patients with peripheral neuropathy.^31^

Yet another significant aspect is that oxidative stress is a fundamental problem of metabolic syndrome. Oxidative stress causes IR in the peripheral tissues by affecting various facets in insulin receptor signal transduction. This culminates in decreased expression of the GLUT4 transporter in the cellular membrane. We also believe that various yogic interventions may be directly beneficial in rejuvenating cells of the pancreas by attenuating oxidative stress. This could lead to an increase in utilization and metabolism of glucose in the peripheral tissues, liver, and adipose tissues.^32^

Enhanced blood supply to the organs holds the key. This is linked to insulin receptor expression in muscles, which in turn increases glucose uptake by muscles and thereby reduces blood sugar.^33^ The improvement in the lipid profile following yoga could be attributed to the increased activity of hepatic lipase and lipoprotein lipase at cellular level. This in turn increases uptake of TAG by adipose tissues.^34^ It is believed that yogic intervention could change the metabolic profile and effect elimination of stress, as stated by previous workers.^35^,^36^

An earlier study has revealed that the practice of Raja yoga meditation could lower serum cholesterol.^34^ The beneficial effects of yoga in alleviating dyslipidemia have been investigated.^37^,^38^

The beneficial effects of yoga as observed in this study are promising and hence in the long-term would bring proper control of blood sugar and stabilize lipid profile level. However, for various reasons, individuals with diabetes often cannot sustain the levels of recommended physical activity, and hence there is an inherent issue with compliance.

We do believe that the stretching of the body during yoga *asanas* could rejuvenate the islet cells, thus increasing insulin secretion and hence correcting the impaired insulin secretion in chronic diabetes.^33^ However, we emphatically state that the protocol developed by us needs to be further taken up for future studies in a comprehensive manner to evaluate short- and long-term benefits of yoga on DM.

It is believed that the conglomerate of psycho- neuro-endocrine and immune mechanisms has holistic effects on diabetes control. Earlier, studies had revealed that parasympathetic activation and the associated anti-stress mechanisms could help improve overall metabolic and psychological profiles, besides enhancing insulin sensitivity, and improving glucose tolerance and lipid metabolism.^39^ We do believe that yoga therapy reduces blood glucose levels by enhancing insulin sensitivity and helps in the management of comorbid disease conditions associated with DM, thereby resulting in significantly improved clinical outcomes. The effect of physical exercise on blood lipid profile has been well documented. Physical activity and HDL appear to be linked through HDL’s role in triglyceride metabolism.^40^ Further studies are needed to investigate the LDL-lowering effect of yoga therapy. Our laboratory is presently pursuing research that includes the effect of yoga therapy in DM as evidenced by reliable markers of oxidative stress, anthropometry, and gene polymorphism of adiponectin, since oxidative stress and adiponectin levels are intrinsically linked to IR. We expect to receive the results of this new study in the coming months.

### Novelty

Prescribed integrated yoga therapy protocol is simple to practice, requires little space, and can be practiced at one’s own convenience in the comfort of the home, at least 2 hours after a full meal. As an adjuvant, non-invasive therapy could help individuals with DM to delay the occurrence of micro- and macro-vascular complications such as retinopathy, neuropathy, nephropathy, and ischemic heart disease.

## CONCLUSION

The results of the present study are encouraging, and it is concluded that integrated yoga therapy as an adjuvant and non-invasive therapy is ideal for people with diabetes. The integrated yoga therapy protocol developed at our institute with a mild set of *asanas* and *pranayama* techniques could be practiced easily and could have beneficial effects in individuals with diabetes.

### Limitations

Due to the small sample size a descriptive analysis was carried out. Age- and sex-matching were not possible. Direct homogeneous assay was not available for performing low-density lipoprotein estimation.

### Future Scope

Yoga therapy is a non-invasive and viable lifestyle modification that may be practiced safely by people with diabetes. Yoga therapy, in association with standard/rational medication, could effectively delay the complications of DM. In the future, the effect of standardized yoga therapy should be considered in the light of gene polymorphisms associated with insulin sensitivity and insulin resistance, which would open newer vistas in personalized medicine. Yoga therapy thus could be considered as an effective therapeutic modality. More studies on yoga intervention in prediabetes, with inputs from lifestyle modifications, could open new horizons in non-invasive medicine based on complementary and adjuvant yoga therapy.

## Abbreviations

BMI: body mass index
DM: diabetes mellitus
FBG: fasting blood glucose
HbA1c: hemoblobin A1c
HDL: high-density lipoprotein
HOMA-IR: homeostatic model assessment for insulin resistance
IR: insulin resistance
PPBG: post-prandial blood glucose
QC: quality control
SBV: Sri Balaji Vidyapeeth
T2D: type 2 diabetes mellitus
WHO: World Health Organization
